# The *pag* Gene of pXO1 Is Involved in Capsule Biosynthesis of *Bacillus anthracis* Pasteur II Strain

**DOI:** 10.3389/fcimb.2017.00203

**Published:** 2017-05-26

**Authors:** Xudong Liang, Jin Zhu, Zhongzhi Zhao, Feng Zheng, Huijuan Zhang, Jianchun Wei, Yon Ji, Yinduo Ji

**Affiliations:** ^1^State Key Laboratory for Infectious Disease Prevention and Control, National Institute for Communicable Disease Control and Prevention, Chinese Center for Disease Control and PreventionBeijing, China; ^2^Huadong Medical Institute of BiotechniquesNanjing, China; ^3^Department of Veterinary and Biomedical Sciences, College of Veterinary Medicine, University of MinnesotaSt. Paul, MN, United States

**Keywords:** *Bacillus anthracis*, pXO1 and pXO2, capsule biosynthesis, *pag* gene, *acpA* and *acpB*

## Abstract

The poly-γ-D-glutamic acid capsule and anthrax toxins are major virulence factors of *Bacillus anthracis*. Genes responsible for capsule biosynthesis are located on pXO2, whereas genes encoding the toxins, which are composed of edema factors, lethal factors, and protective antigens (PA), are located on pXO1. In this study, we found that the *pag* null mutation not only eliminated the production of the protective antigen, it also eliminated the ability of the *B. anthracis* Pasteur II strain to form capsules. qPCR analysis revealed that the deletion of *pag* decreased the transcription levels of the *capABCD* operon and its regulatory genes *acpA and acpB*. The introduction of the *acpA* or *acpB* plasmid complemented the effect of the *pag* null mutation on capsule formation. Taken together, the above results suggest that PA probably affects capsule biosynthesis by altering the expression of *acpA and acpB*. In addition, we found that the deletion mutation of *pag* remarkably attenuated bacterial pathogenicity in a mouse model of infection. Our results indicate that besides encoding the protective antigen, the *pag* gene of pXO1 is also involved in the modulation of capsule biosynthesis. Our findings provide new insight into the regulation mechanisms of capsule formation in *B. anthracis* Pasteur II strain.

## Introduction

Anthrax is an anthropozoonosis caused by *Bacillus anthracis*. Clinical manifestations of this disease include cutaneous, gastrointestinal, or respiratory infections. Sporadic prevalence in humans and animals has led to small outbreaks of this disease worldwide (Liang, 1995). As a biological warfare agent, *B. anthracis* has the potential to cause devastating societal and economic losses. Therefore, understanding the pathogenic mechanism of *B. anthracis* remains relevant and has been the focus of research in recent years (Moayeri et al., 2015).

The virulence factors of *B. anthrac*is, including anthrax toxins and the antiphagocytic polyglutamic capsule, are encoded by the pXO1 and pXO2 plasmids, respectively. The loss of these two plasmids leads to the loss of toxins and capsule formation, resulting in a loss of pathogenicity (Mikesell et al., 1983; Uchida et al., 1986). The pathogenicity of *B. anthracis* toxins and the capsule has been intensively explored in various animal models (Smith and Keppie, 1954; Stanley and Smith, 1961). Toxicity was observed only when the protective antigen (PA) was present with either the edema factor (EF) or lethal factor (LF), indicating that PA is a toxin mediator (Fish et al., 1968; Remmele et al., 1968). These toxin factors are encoded by *pag cya*, and *lef*, respectively, and are regulated by the *atxA* gene on pXO1 (Okinaka et al., 1999).

A 63-kDa fragment of PA, PA63, oligomerizes into heptamers or octamers to form the LF/EF binding sites (Leppla, 1988; Singh et al., 1989, 1999). Upon binding to the cell receptor, the PA binds LF and EF. After endocytosis and endosomal acidification, LF and EF are transferred into the cytoplasm to access their targets, including the mitogen-activated protein kinase kinase (MAPKK) family and ATP (Abrami et al., 2003). The lethal toxin inhibits the MEKs signaling pathway, and in turn, prevents the release of chemotactic factors and cytokines, which can induce toxic shock and is the reason behind the high mortality rate of *B. anthracis* infections (Milne et al., 1994; Gordon et al., 1995; Erwin et al., 2001; Abrami et al., 2003).

The poly-γ-D-glutamic acid capsule is the other key virulence factor and is synthesized by the *capBCAD* operon on pXO2 (Makino et al., 1989). Besides the anthrax toxin activator, *atxA*, encoded on pXO1, the expression of toxin genes, including *cya, lef*, and *pag* on pXO1, are affected by the expression levels of the capsule genes on pXO2, as well as increased carbon dioxide (CO_2_) and bicarbonate (${\text{HCO}}_{3}^{-}$) levels during the time the pathogen enters the host (Bartkus and Leppla, 1989; Koehler et al., 1994; Sirard et al., 1994). Both *atxA* and *acpA* genes modulate the toxins and capsule production, respectively (Uchida et al., 1993; Guignot et al., 1997; Hoffmaster and Koehler, 1997; Uchida et al., 1997). Our previous studies clearly demonstrated that the *B. anthracis* Pasteur II vaccine strain possesses low copy numbers of the pXO1 plasmid, which dramatically affect the expression levels of toxin genes (Liang et al., 2016).

It is well established that the *acpA* gene on pXO2 is responsible for controlling capsule synthesis in *B. anthracis*. However, the reason why some *B. anthracis* strains harbor pXO2 but lack capsule production is unclear (Ezzell and Welkos, 1999). In this study, we demonstrated that the *pag* gene on pXO1 plays an important role in capsule biosynthesis of the *B. anthracis* Pasteur II strain by using gene deletion and complementation studies. Furthermore, we found that PA affects the transcription of *acpA* gene, which in turn mediates capsule production.

## Materials and methods

### Bacterial vaccine strain

*B. anthracis* vaccine Pasteur II strain was kindly provided by the Institute of Lanzhou Biological Products in China.

### Construction of gene mutants and overexpression strains

#### Construction of the *pag* gene promoter deletion strain

We designed primers directed to the upstream region of the *pag* gene promoter: 141819F_BamHI and 143181R_crossover (Table 1) and introduced a *Bam*HI endonuclease recognition site into the 141819F_BamHI primer. An upstream homology arm approximately 1,360 bp in length was amplified by PCR with wild-type strain DNA used as template. The downstream primers were 143779F_crossover and 144559R_Bgl with a *Bgl*II site introduced into the 144559R_Bgl primer (Table 1). A downstream homology arm approximately 800 bp in length was amplified. Since parts of the 143181R_crossover and 143779F_crossover sequences were complementary to each other, we simply mixed the upstream and downstream PCR products and amplified a 2.2-kb fragment composed of an sequences of the upstream and downstream arms of the *pag* gene using the 141819F Bam and 144559R Bgl primers. This amplicon was double-digested with *Bam*HI and *Bgl*II and cloned into the *Bam*HI/*Bgl*II site of pMAD, to produce the recombinant plasmid pag-pMAD. The recombinant plasmid was electroporated into the Pasteur II strain. Integrons containing pag-pMAD were obtained at 42°C and subsequently cultured at 30°C to promote gene replacement. The target gene deletion strain, PasteurII-pagpromoterKO, was identified from all erythromycin sensitive strains by using PCR and confirmed by sequencing.

**Table 1:** **Oligonucleotides used in this study**.

**Gene or Primer**		**Sequence (5′–3′)**
16SrRNA	For	CTACAATGGACGGTACAA
	Rev	CTACAATCCGAACTGAGAA
*gerXB*	For	GAAGAATGGCGACTTGTA
	Rev	ATAACGAGTGATATACGAATGA
*pag*	For	TACAAGTGCTGGACCTAC
	Rev	CCGTATATCCTTCTACCTCTAA
*atxA*	For	GTAGCGTCTATAACCTCAG
	Rev	TTGCTGTCTGTGGTAATAG
*capA*	For	GTATTACCTCTTATCGCAGTTAT
	Rev	ACCATCGTCATCGTCAAT
*capB*	For	TTGTGAATGTATGGCAGTT
	Rev	TATGGAATGGTAGCAGTGA
*capC*	For	GGAGTTACACTGAGCCTTAT
	Rev	TGAATCTTGAAACACCATACG
*capD*	For	ATTGCTTAGTGTATTAGTTGA
	Rev	AAGAATGAGAATGGTGATG
*acpA*	For	GTCTATGGAATGATTGAGTA
*acpB*	Rev	CATCGGGAATATCTGTTAA
	For	TTGGGCAGAGTATCTAGCTG
	Rev	ATCGCGTTCTGTAGGGATAG
*spoOA*	For	TCTACTGTTGTTGCTGAT
	Rev	GAGTCATATTCGTCAAGTG
*rpoB*	For	CCA ACAGTAGAAATGCC
	Rev	AATTTCACCAGTTTCTGGATCT
*abrB*	For	GTTACCGTCAGATACTTC
	Rev	GATGAATTAGGTCGTGTAG
141819F_BamHI		GGAGGATCCTTGTGTTTTATGCCATAATAG
143181R_crossover		TAACACTTTTCGTTTAAACTTATGTGAAACAAAGAT
143779F_crossover		ATCTCTTTGTTTCACATATGAAAAAACGAAAAGTGTTA
144559R_Bgl		AGAAGATCTCTCTCTTTTCGAAATCACTCTGTACGG
PApr-175SacF		ATCGCCGCGGTTTTTTCTAAATATACAGTGTAAG
PA-RSacII		CAGCCGCGGTTATCCTATCTCATAGCCTTTT
PApr-385SacF		ATCGCCGCGGGTTTCAAGGTACAATAATTATGG
PA-RSacII		CAGCCGCGGTTATCCTATCTCATAGCCTTTT
PApr-432SacF		CAGCCGCGGTTCTTTCAGGTTGTTTTTGGGT
PA-RSacII		CAGCCGCGGTTATCCTATCTCATAGCCTTTT
142918SacF		TATTAGTAGCACAGTTTTTG
PApr-604SacR		ACACAGAAGCTGTTTTTGAAG
143120F_BamHI		GGAGGATCCTTCTTTTAATAAGGAGCTGCC
143898R_crossover		CTCTCTCTCTCTCACTAGGATTAATCCTGGGAACTTGATTCTGAT
145987F_crossover		AATCAAGTTCCCAGGATTAATCCTAGTGAGAATGGG
146648R_Bgl		AGAAGATCTCCACTTTTATCTTTTCCAACAG
PA-FSacII		CAGCCGCGGTGTAAAACAGCCTTAATAGTTG
PA-RSacII		CAGCCGCGGTTATCCTATCTCATAGCCTTTT
*acpA*OE-p40-F		CAGCCGCGGATGGAAAAAGATATTAGCCGAA
*acpA*OE-p40-R		CAGCCGCGGCTAACCATCTTGTAAATCTAGATA
*acpA*promo_F		ATTTCGTAGGGCTTGCATATT
acpApromo_R		CAAATCAATTTTTCGGCTAAT
acpBOE-p40-F		CAGCCGCGGAATTGGATATTTTTATTGGAAATG
acpBOE-p40-R		CAGCCGCGGCTAACCATCTTGTAAATCTAGATA

#### Construction of *pag* gene complementary strain

**Figure 1:**
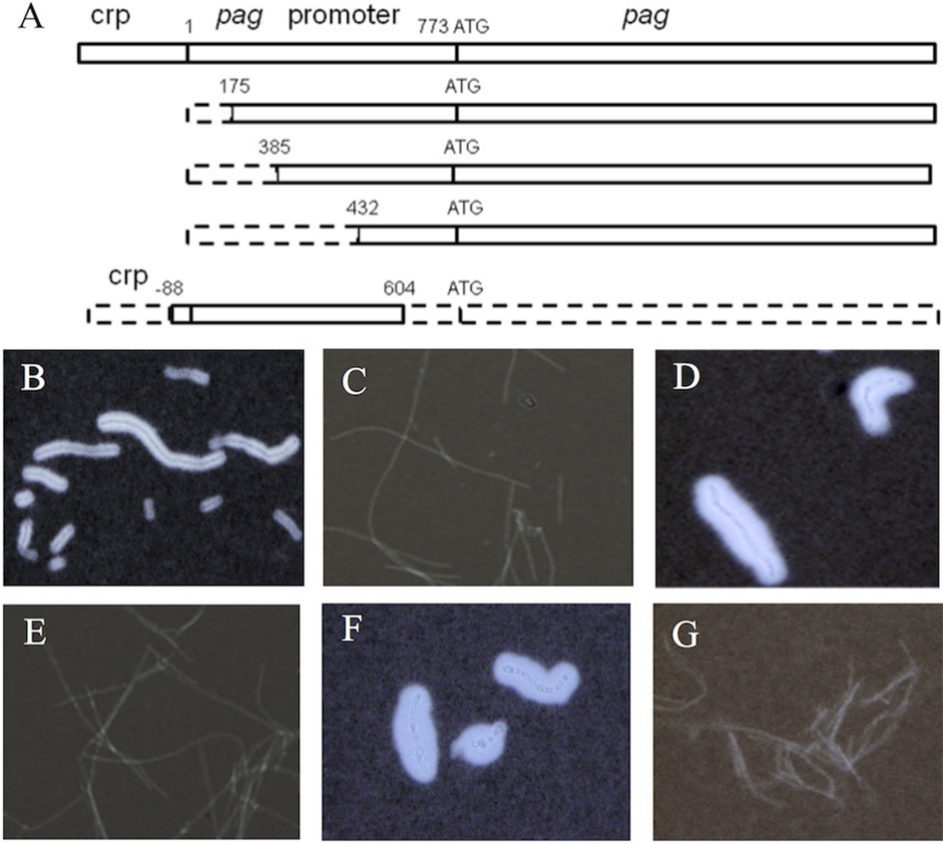
**Effect of the *pag* gene on capsule formation of Pasteur II strain**. Diagram of the *pag* promoter region and *pag* gene location **(A)**. The dashed boxes indicate truncated regions; the solid boxes indicate untruncated regions. Microscope images of India ink stained Pasteur II strain **(B)**, PasteurII-pag promoter KO strain with the longest fragment deletion of the *pag* promoter region **(C)**, PasteurII-pag promoterKO-CO strain **(D)**, PasteurII-pagKO strain **(E)**, PasteurII-pagKO-CO strain **(F)**, and negative control, Sterne strain without capsule formation **(G)**. The images are representative of 3 independent experiments.

Primers directed to the upstream region of the *pag* gene promoter were designed using Oligo 6.0 software. Four primer sets, PApr-175SacF and PA-RSacII, PApr-385SacF and PA-RSacII, PApr-432SacF and PA-RSacII, 142918SacF and PApr-604SacR (Table 1), and high-fidelity polymerase Pyrobest (Takara, Dalian, China) were used to amplify different sizes of fragments covering *pag* and its promoter region on pXO1 (Figure 1). After initial denaturation at 95°C for 5 min, reactions were performed for 30 cycles, with each cycle consisting of denaturation (95°C for 30 s), annealing (55°C for 30 s), and extension (72°C for 2 min). A 2.3-kb fragment was amplified, purified, digested with *Sac*II, and cloned into the *Sac*II site of the expression vector pFF40. The recombinant plasmid was transformed into *Escherichia coli* (DH5α), and the positive clones were screened by PCR and identified by double digestion. The recombinant plasmid was electroporated into the PasteurII-pag promoterKO strains. The positive clone was sequenced to ensure no mutations. The positive strain was designated PasteurII-pagpromoter-KO-CO.

#### Construction of *pag* gene deletion strain

The *pag* gene deletion strain was obtained using methods similar to those described above. We designed primers to the upstream region of *pag*: 143120F_BamHI and 143898R_crossover and introduced a *Bam*HI site into the 143120F_Bam primer (Table 1). An upstream homology arm of approximately 800 bp was amplified by PCR using wild-type strain DNA as template. A downstream homology arm approximately 700 bp in length was amplified using the primers: 145987F_crossover and 146648R_Bgl with a *Bgl*II site added to the 146648R_Bgl primer (Table 1). The fragment containing both 143898R and 145987F arms was amplified by crossover PCR, double digested with BamHI and BglII, and cloned into the pMAD plasmid, to produce the recombinant plasmid pag-pMAD. The recombinant plasmid was electroporated into the PasteurII strain. The target gene deletion strain PasteurII-pagKO was identified from all erythromycin sensitive strains using PCR (Supplementary Figure 1) and confirmed by sequencing.

#### Construction of *pag* gene complementary strain

The *pag* gene on pXO1 was replaced using methods similar to those described above. Primers directed to *pag* were designed using Oligo 6.0 software with *Sac*II restriction sites: PA-FSacII and PA-RSacII. The PCR product was digested with *Sac*II and cloned into the *Sac*II site of the expression vector pFF40. The recombinant plasmid was transformed into *E. coli* DH5α and the positive clones were screened by PCR and identified by double digestion. The resultant plasmid was electroporated into PasteurII-pagKO. The positive clone was selected with kanamycin and designated PasteurII- pagKO-CO after identification by PCR (Supplementary Figure 2) and confirmation by sequencing.

#### Construction of *acpA* or *acpB* gene complementation strain in *pag* knockout mutant

Similar methods were applied to construct the *acpA* gene complementation strain. The primers used were *acpA*OE-p40-F and *acpA*OE-p40-R (Table 1). The PCR product was digested with *Sac*II and cloned into the *Sac*II site of pFF40. The recombinant plasmid was transformed into *E. coli* DH5α and the positive clone was identified by double digestion. The resultant plasmid was electroporated into PasteurII-pagKO, confirmed by DNA sequencing, and designated PasteurII-pagKO-acpA and PasteurII-pagKO-acpB

### RNA isolation and quantitative real-time PCR analysis (qPCR)

Overnight cultures of strains tested were incubated in LB medium and grown to mid-log phase (OD_600_ = 0.6) at 37°C with shaking. The cells were harvested by centrifugation, and total RNA was purified using the RNeasy Mini Kit (Qiagen) according to the manufacturer's instructions. The primers used for each target are listed in Table 1. cDNA was synthesized from 1 μg RNA using Superscript III reverse transcriptase (Invitrogen) and random hexamers following the manufacturer's protocol. The cDNA was diluted 5-fold and was amplified by qPCR. The gene expression levels as indicated by CT values were compared among the strains tested. 16S rRNA was used as a reference gene for normalization. The qPCR was repeated three times.

### Western-blotting analysis

Strains were cultured overnight, and samples of the supernatant were collected and filtered through 0.22 μm-pore-size syringe filters. An aliquot of 5 ml of the filtered supernatant was mixed with an equal volume of 95% ethanol and incubated at 4°C overnight, followed by centrifugation. The precipitate was dried at 22–25°C and resuspended in 50 μl PBS. Following the addition of the loading buffer, the sample was heated to 100°C for 5 min. Twenty microliter of each sample (total protein content 5 μg) was loaded onto a 12% SDS-PAGE gel. The samples were then transferred onto a nitrocellulose membrane (Bio-Rad). The membranes were blocked for 1 h with TBS-T (20 mm Tris base, 137 mm NaCl, 0.1% Tween20) containing 5% dry milk at room temperature. The membranes were then incubated with either an anti-protective antigen (PA) antibody or anti-LF antibody (Thermo Scientific), and then diluted 1:2000 in TBS-T-5% milk overnight at 4°C. Membranes were washed in TBS-T and incubated with HRP-conjugated goat anti-mouse IgG secondary antibody diluted 1:4,000 in TSB-T-5% milk for 1 h at room temperature. After the same washing procedure, proteins were detected using the ECL Western Blot Substrate (Pierce) according to the manufacturer's instructions.

### Gel mobility shift DNA binding assay

To explore whether protective antigen (PA) is able to directly regulate the transcription of *acpA* gene, we performed a gel shift assay. A DNA fragment of the upstream region of *acpA* was amplified by PCR using biotin-labeled primers: *acpA*promo_F and acpApromo_R (Table 1). The amplified DNA fragments were purified using the QIAquick PCR Purification kit (Qiagen) according to the manufacturer's instructions. DNA binding and electrophoresis were performed as described (Yang et al., 2015). Briefly, commercial-grade protective antigen protein (Listbiological Laboratories, Inc.) was added to a 20 μl mixture containing 0.2 pmol biotin-labeled DNA, 1 mg of poly(dI-dC), 25 mM NaH_2_PO_4_ (pH 8.0), 50 mM NaCl, 2 mM MgCl_2_, 1 mM dithiothreitol (DTT), 10% glycerol, and 0.1 mM EDTA. The concentrations of PA protein used were 0.4, 1, 2, 4, and 8 μM (corresponding to 0.2, 0.5, 1, 2, and 4 μg, respectively). Unlabeled DNA fragments of the promoter region were added into the reaction at a 120-fold concentration over the labeled probe to inhibit non-specific binding. Bovine serum albumin (BSA) was added to prevent non-specific protein binding. The binding reaction was initiated by the addition of PA, and the reaction mixture was incubated at room temperature for 25 min. Samples were then loaded onto a 6% native polyacrylamide gel (acrylamide-bisacrylamide [29:1] in 0.5 × Tris-borate-EDTA [TBE] buffer). Electrophoresis was performed for 3 h at 4°C with 7 V cm^−1^, and the gels were electroblotted onto nylon membranes in 0.5 × TBE at 300 mA for 90 min at 4°C. After cross-linking the DNA-protein hybrids with UV radiation, the membranes were hybridized with stabilized streptavidin-HRP conjugate (Thermo Scientific) followed by incubation with DAB substrate at room temperature for 20–30 min to optimize signal intensity.

### Microscopy analysis of capsule with india ink staining

The *B. anthracis* isolates were incubated in a LB medium with 0.9% NaHCO_3_ under 5% CO_2_ for 8 h. The bacterial cells were stained with India ink and were observed under a microscope. The Sterne strain without capsule formation was used as negative control. The bacterial capsule is visible in India ink because it excludes the ink particles. Each experiment was repeated at least three times.

### Mouse infection

Six to eight week old BALB/C male mice (18–20 g) were used for the assessment of pathogenicity of the Pasteur II *pag* knockout mutant. Briefly, mice were randomly divided into two groups (*n* = 6) and injected with 10^4^ CFU ml^−1^ Pasteur II and PasteurII-pagKO, respectively, via subcutaneous injection to the inner thigh. The mice were observed continuously for 60 min, and afterwards once every 12 h. All experiments involving animals were performed in accordance with the protocols approved by the Animal Care and Use Committee of the National Institute of Allergy and Infectious Diseases, National Institutes of Health, China. Significance in animal survival was determined by Fisher exact test.

## Results

### The mutation of *pag* promoter or *pag* gene eliminates capsule formation

The expression levels of the capsule genes on pXO2 affect the expression of toxin genes, including *cya, lef*, and *pag* on pXO1 (Barkus and Leppla, 1989; Koehler et al., 1994; Sirard et al., 1994), indicating the interplay between the two plasmids. During our spontaneous mutagenesis studies, we found that the *pag* gene on pXO1 might affect the expression of the capsule genes on pXO2. To determine the role of *pag* in capsule production, we deleted the *pag* gene promoter region in the pXO1 plasmid (Figure 1A). Compared to the Pasteur II control (Figure 1B), the *pag* promoter knockout mutant, PasteurII-pagpromoterKO (generated with PApr-175SacF and PA-RSacII), produced dramatically less capsule (Figure 1C), which is similar to the capsule negative control Sterne strain (Figure 1G). To verify if the capsule deficiency was caused by the deletion of the *pag* promoter, we subcloned fragments of the *pag* gene and promoter into the *pag* promoter knockout strain for functional complementation assays (Figure 1A). Our results show that only the strains complemented with fragments encoding the entire *pag* gene restored capsule synthesis (Figure 1D), suggesting that *pag* is involved in capsule formation.

To confirm the role of *pag* gene in capsule formation, we deleted the entire *pag* gene. Similar to the capsule negative control Sterne strain (Figure 1G), the *pag*-deletion strain displayed capsule deficiency (Figure 1E). The morphology of PasteurII-pagKO deletion strain showed hair-like, dry, smooth, and non-sticky colonies, which was significantly different from the morphology of the Pasteur II control (data not shown). To further confirm the effect of *pag* on capsule formation, we conducted the complementation experiment by introducing pFF40 carrying the *pag* gene into the *pag* deletion strain. The complementary strain completely recovered the capsule formation, with thicker capsule morphology and increased capsule substance as compared to the Pasteur II strain (Figure 1F).

### The deletion of *pag* promoter or *pag* entire gene abolishes the PA production

**Figure 2:**
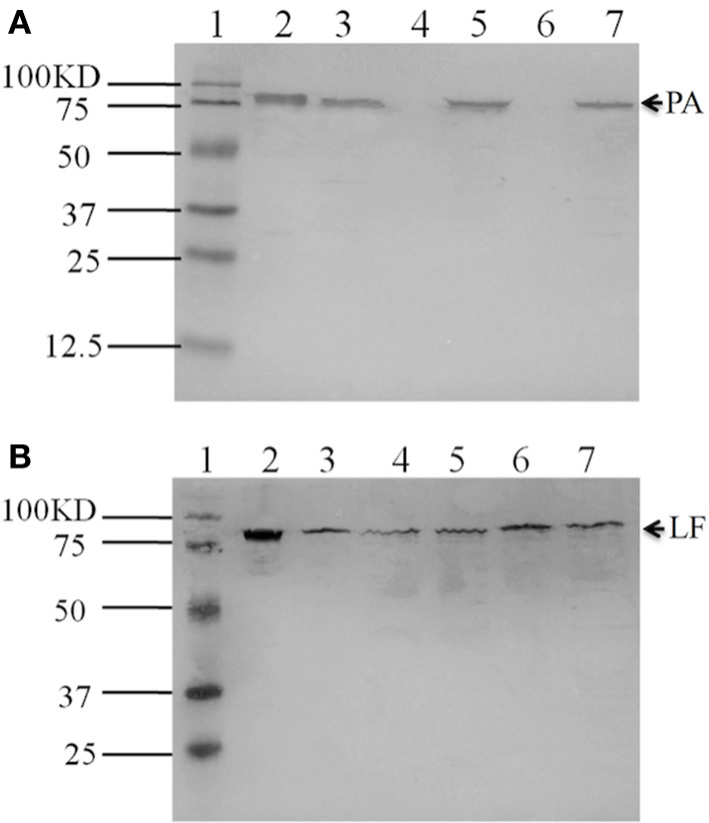
**Western blot detection of PA and LF expressed in variant strains**. PA was detected by hybridization with anti-PA antibody **(A)**. Lane 1, protein molecular size marker; Lane 2, control PA (100 ng); Lane 3, Pasteur II; Lane 4, PasteurII-pag promoter-KO strain; Lane 5, PasteurII-pag promoter-KO-CO strain; Lane 6, PasteurII-pagKO strain; Lane 7, PasteurII-pagKO-CO strain. LF was detected by hybridization with anti-LF antibody **(B)**. Lane 1, protein molecular size marker; Lane 2, control LF (100 ng); Lane 3, Pasteur II; Lane 4, PasteurII-pag promoter-KO strain; Lane 5, PasteurII-pag promoter-KO-CO strain; Lane 6, PasteurII-pagKO strain; Lane 7, PasteurII-pagKO-CO strain.

To elucidate whether the capsule defect phenotype of *pag* null knockout mutants is a result of the lack of PA production, we performed Western blot assays. The results showed that the expression of *pag* was not detected in either the *pag* promoter or the entire *pag* gene null mutants (lane 4 and lane 6 in Figure 2A), whereas the *pag* null mutation did not affect LF production (lane 4 and lane 6 in Figure 2B). This indicates the specific effect of the *pag* promoter or *pag* null mutation on PA production.

Taken together, the above results demonstrated that the PA protein is involved in capsule formation.

### The deletion of *pag* alters the transcription of virulence genes located on both pXO1 and pXO2 plasmid

To elucidate the potential mechanism of PA's involvement in capsule formation, we examined the impact of the *pag* deletion on the transcription of selected virulence genes located on pXO1 and pXO2. The total RNA was extracted from PasteurII-pag KO and the original Pasteur II control, and was analyzed by qPCR. In the *pag* deletion strain, no *pag* expression was detected, as had been expected (Table 2). However, the expression of virulence genes *gerXB* and *atxA*, which are located in plasmid pXO1, showed a 2-fold increase and 2-fold decrease, respectively, in PasteurII-pag KO compared with that in Pasteur II control. In addition, the expression levels of genes responsible for capsule synthesis, including *capA, capB, capC*, and *capD*, decreased 4-fold in the PasteurII-pag KO strain compared with those in Pasteur II control, whereas the expression level of both *acpA* and *acpB* decreased 8-fold (Table 2). Surprisingly, the *pag* deletion also altered the transcription of the *spoA, abrB*, and *rpoB* genes located in chromosomal DNA (Table 2).

**Table 2:** **qPCR analysis of the effect of *pag* on transcription of virulence genes**.

**Gene**	Fold change (PasteurII-pag KO/PasteurII)^*^
**GENES ON pXO1**	
*gerXB*	+4
*pag*	N/A
*atxA*	−2
**GENES ON pXO2**	
*capA*	−4
*capB*	−4
*capC*	−4
*capD*	−4
*acpA*	−8
*acpB*	−8
**GENES ON CHROMIC DNA**	
*spoOA*	+2
*abrB*	−8
*rpoB*	−8

* *qPCR was repeated 3 times with similar results*.

### The introduction of constitutive *acpA* or *acpB* expression system eliminates the effect of deleted *pag* on capsule formation

**Figure 3:**
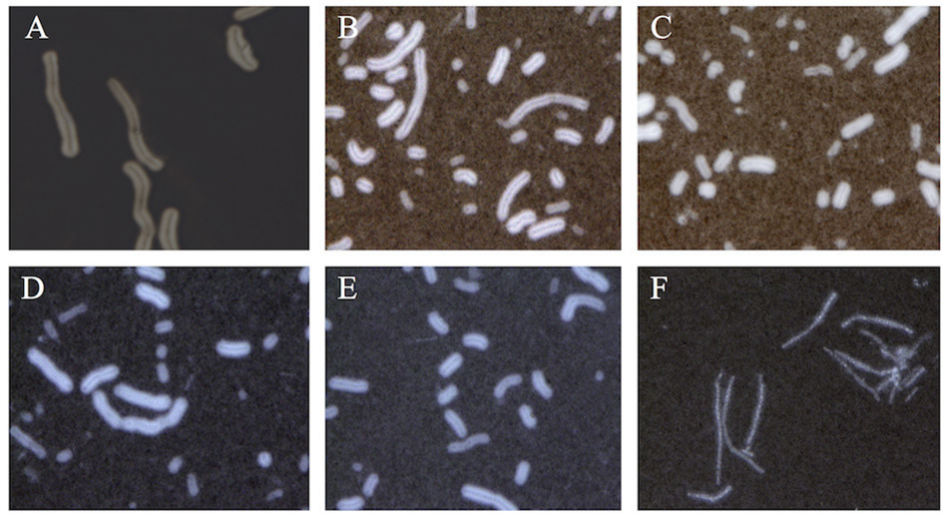
**Complementation effect of AcpA and AcpB on capsule formation in the *pag* null mutants**. Microscope images of India ink stained Pasteur II strain **(A)**, *acpA* complementation strain in the *pag* promoter deletion mutant **(B)**, and *acpA* complementation strain in the *pag* null mutant **(C)**, *acpB* complementation strain in the *pag* promoter deletion mutant **(D)**, *acpB* complementation strain in the *pag* null mutant **(E)**, and negative control Sterne strain without capsule formation **(F)**. The images are representative of 3 independent experiments.

Our qPCR results suggested that PA is probably involved in the capsule formation by mediating the transcription of *acpA* and *acpB*. To test it, we constructed a constitutional *acpA* or *acpB* expression strain in the *pag* promoter and *pag* gene knockout mutant, respectively. Compared to the Pasteur II control (Figure 3) and the *pag* null mutants (Figures 1C,E), the ability of capsule formation was fully restored in the *pag* promoter, the entire *pag* gene knockout mutant carrying on the constitutive *acpA* expression system (Figures 3B,C), or carrying on the constitutive *acpB* expression system (Figures 3D,E). To explore the possibility of whether PA directly affects the *acpA* transcription by binding to the promoter region of *acpA*, we conducted a gel mobility shift assay using purified recombinant PA, and labeled the *acpA* gene promoter region. No shift of the DNA/protein complex band was observed (data not shown), indicating that the PA protein did not directly mediate the *acpA* transcription.

### The deletion of *pag* attenuates the virulence of the *B. anthracis* Pasteur II strain

**Figure 4:**
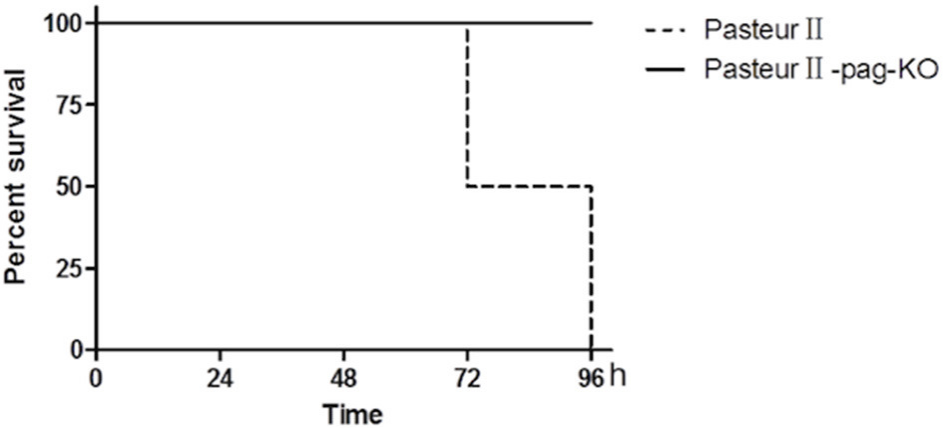
**Effect of *pag* on pathogenicity of *B. anthracis* in BALB/c mouse model of infection**. Six mice each were infected with either Pasteur II (dashed line), or PasteurII-pagKO strain (solid line), Percent of surviving mice shown as a function of time in hours post-infections. The difference of animal survival was statistically analyzed using Fisher exact test. *P* < 0.01.

The role of PA in pathogenicity is controversial in different mouse models of infection (Welkos et al., 1993; Heninger et al., 2006; Moayeri et al., 2015). In this study, we examined the effect of *pag* on the pathogenicity of *B. anthracis* Pasteur II strain in the BALB/c mice model of subcutaneous infection. All BALB/c mice infected with PasteurII-pagKO survived for at least 96 h, whereas 50% (3 of 6) of the mice infected with the Pasteur II control strain died by 72 h, and 100% of them died by 96 h after infection. The mortality of mice did not increase when infected with more than 2-fold higher amounts of the *pag* knockout mutant than wild type control in subsequent experiments. The results indicated that the *pag* gene is an important virulence factor for *B. anthracis* Pasteur II strain in subcutaneous infection (Figure 4).

## Discussion

*B. anthracis* virulence is plasmid-determined, with the anthrax toxin genes on pXO1 and the capsule genes on pXO2. Both plasmids are required for virulence; strains without pXO1 are avirulent as they do not produce anthrax toxins, whereas strains lacking pXO2, such as the Sterne vaccine strain, are greatly attenuated since they do not form capsules (Koehler et al., 1994; Hoffmaster and Koehler, 1999). Different regulators of *B. anthracis* control the toxin production and capsule formation. *acpA* regulator controls the *capABCD* operon, which is located on pXO2 and is responsible for capsule biosynthesis. In this study, we demonstrated for the first time that the protective antigen protein is also involved in mediating the capsule formation through interaction with *acpA* and *acpB* in the *B. anthracis* Pasteur II strain.

Our results showed that interrupting PA production by either deleting the *pag* promoter or knocking out the *pag* gene eliminated the formation of capsules in the *B. anthracis* Pasteur II strain. Moreover, the expression of *pag in trans* complemented the capacity of capsule formation. These indicate that the PA plays a role in capsule biosynthesis. However, other investigators have shown that the non-toxigenic (pXO1^−^, pXO2^+^) delta Ames strain produces capsule (Welkos et al., 1993; Pomerantsev et al., 2006). This inconsistency is likely due to the different genetic backgrounds between the Pasteur II and Ames strain. Another explanation is that the non-toxigenic Ames strain may still possess the pXO1 plasmid, and as we have demonstrated, there is an existence of the pXO1 plasmid in the Pasteur II strain, and high temperature treatment could not completely eliminate the pXO1 plasmid in *B. anthracis* (Liang et al., 2016).

The impact of the *pag* deletion on the transcription of virulence genes on both pXO1 and pXO2 suggests that PA probably indirectly modulates the transcription of the *capABCD* operon and its regulators *acpA and acpB*. Although deletion of *pag* only generated a 4-fold difference of *cap* transcription levels compared to the control, this suggests a potential accumulative effect of PA protein on capsule formation, as the qPCR analysis provides only a snapshot. Our data showed that the introduction of the episomal *acpA* or *acpB* gene restored capsule formation of the *pag* knockout strain, indicating the critical role of AcpA/AcpB in capsule formation. Drysdale *et al*. reported that an *acpA* null mutation slightly affected the *cap* transcription, whereas an *acpA* and *acpB* double mutation totally eliminated *cap* gene expression (Drysdale et al., 2004). One possible explanation is that the AcpA/AcpB-dependent modulation of capsule formation by PA protein is strain dependent. However, DNA-protein binding analysis revealed that PA could not bind the promoter region of *acpA*. Taken together, this data demonstrates that PA is involved in regulating capsule formation through interaction with the capsule regulators *acpA* and *acpB* gene of Pasteur II strain by an unknown mechanism.

PA can bind and then facilitate EF and LF entry into cells to exert their toxic effects without itself showing any toxic effect, and is an effective protective antigen and strong immunogen. EF is an adenylate cyclase that increases cyclic AMP (cAMP) levels in host cells. The increased cAMP activates PKA and cAMP-activated exchange protein (Epac) signaling, which inhibits the movement and phagocytosis of macrophages (Yeager et al., 2009). LF is the principal virulence factor of anthrax toxin. A high dose of LF can cause macrophage schizolysis, while a small dose of LF can induce macrophage apoptosis by altering cell membrane permeability, depleting the mitochondrial electric potential, or fragmenting DNA. The pXO1 plasmid plays a key role in the regulation of protein function in *B. anthracis* (Popov et al., 2002; Park et al., 2007). Consistent with previous studies, our results also showed that the *pag* knockout strain lacked the expression of PA protein and attenuated the virulence of the *B. anthracis* Pasteur II strain. However, other groups have reported that the *pag* null mutation had no effect on LD50 values of *B. anthracis* Ames strain (Welkos et al., 1993; Heninger et al., 2006). These contradictions may result from the different genetic background between the Pasteur II strain and other *B. anthracis* strains used by other research groups. It is possible that the mouse strain and route of infection also affects the susceptibility to *B. anthracis*, as we used subcutaneous inoculation of bacteria in the inner thigh of BALB/C mice, whereas Welkos infected CBA/J and A/J mice via subcutaneous or intraperitoneal inoculation (Welkos et al., 1993), and Heninger utilized BALB/C mice through intratracheal infections (Heninger et al., 2006).

Our results indicate that PA indirectly controls the transcription and synthesis of capsule genes by affecting the expression of the capsule-regulating genes, *acpA and acpB*. The regulated capsule genes are located on pXO2, while PA encoding gene *pag* is located on pXO1 in *B. anthracis*. However, we found that the *acpA* gene on pXO2 had no influence on the expression of the toxin genes (data not shown), which is consistent with previous reports (Bourgogne et al., 2003; Drysdale et al., 2005). AtxA has been shown to regulate capsule through *acpA* and *acpB* (Uchida et al., 1997), and *pag* affected the *atxA* transcription (Table 2). Thus, it is highly possible that pag affects capsule through *atxA*. In addition, we found that the deletion of *pag* also decreased the transcription of and the chromosomal genes, including *spoA, abrB*, and *rpoB*. This suggests that other factors may act as intermediate regulators in the interaction with *pag* and influence capsule synthesis. Nevertheless, our data suggests that the *pag* gene is an important factor in the expression of *B. anthracis* virulence factors, including toxins and capsule formation.

In conclusion, to our knowledge, this study is the first to demonstrate that the *pag* gene is critical to capsule production in the *B. anthracis* Pasteur II strain. The pXO1 plays a role in the regulation of capsule formation, and is thus a determinant of *B. anthracis* pathogenicity. The results of our study provide new insights into the mechanisms underlying attenuated virulence in the *B. anthracis* Pasteur II vaccine strain. These findings will potentially guide the development of more efficient vaccines to prevent anthrax.

## Author contributions

Conceived and designed the experiments: XL. Performed the experiments: XL, ZZ, FZ, HZ. Analyzed the data: XL, JZ, JW, YJ, YDJ. Wrote the paper: XL, YDJ.

### Conflict of interest statement

The authors declare that the research was conducted in the absence of any commercial or financial relationships that could be construed as a potential conflict of interest.

## Supplementary material

Supplementary Figure 1 **The deletion of pag gene promoter as indicated by PCR**. Lane 1: DNA molecular marker. Lane 2: PasteurII, and Lane 3: PasteurII- pag promoter-KO.

Supplementary Figure 2 **The deletion of pag gene as indicated by PCR**. Lane 1: DNA molecular marker. Lane 2: PasteurII, and Lane 3: PasteurII- pag KO.

## References

[B1] Abrami L. Liu S. Cosson P. Leppla S. H. van der Goot F. G. (2003). Anthrax toxin triggers endocytosis of its receptor via a lipid raft-mediated clathrin-dependent process. J. Cell Biol. 160, 321–328. 10.1083/jcb.200211018 12551953 PMC2172673

[B2] Bartkus J. M. Leppla S. H. (1989). Transcriptional regulation of the protective antigen gene of *Bacillus anthracis*. Infect. Immun. 57, 2295–2300. 2501216 10.1128/iai.57.8.2295-2300.1989 PMC313445

[B3] Bourgogne A. Drysdale M. Hilsenbeck S. G. Peterson S. N. Koehler T. M. (2003). Global effects of virulence gene regulators in a *Bacillus anthracis* strain with both virulence plasmids. Infect. Immun. 71, 2736–2743. 10.1128/IAI.71.5.2736-2743.2003 12704148 PMC153248

[B4] Drysdale M. Bourgogne A. Hilsenbeck S. G. Koehler T. M. (2004). *atxA* controls *Bacillus anthracis* capsule synthesis via *acpA* and a newly discovered regulator, *acpB*. J. Bacteriol. 186, 307–315. 10.1128/JB.186.2.307-315.2004 14702298 PMC305762

[B5] Drysdale M. Heninger S. Hutt J. Chen Y. Lyons C. R. Koehler T. M. (2005). Capsule synthesis by *Bacillus anthracis* is required for dissemination in murine inhalation anthrax. EMBO J. 24, 221–227. 10.1038/sj.emboj.7600495 15616593 PMC544908

[B6] Erwin J. L. DaSilva L. M. Bavari S. Little S. F. Friedlander A. M. Chanh T. C. (2001). Macrophage-derived cell lines do not express proinflammatory cytokines after exposure to *Bacillus anthracis* lethal toxin. Infect. Immun. 69, 1175–1177. 10.1128/IAI.69.2.1175-1177.2001 11160016 PMC98000

[B7] Ezzell J. W. Welkos S. L. (1999). The capsule of *bacillus anthracis*, a review. J. Appl. Microbiol. 87:250. 10.1046/j.1365-2672.1999.00881.x 10475959

[B8] Fish D. C. Mahlandt B. G. Dobbs J. P. Lincoln R. E. (1968). Purification and properties of *in vitro*-produced anthrax toxin components. J. Bacteriol. 95, 907–918. 4966833 10.1128/jb.95.3.907-918.1968 PMC252109

[B9] Gordon V. M. Klimpel K. R. Arora N. Henderson M. A. Leppla S. H. (1995). Proteolytic activation of bacterial toxins by eukaryotic cells is performed by furin and by additional cellular proteases. Infect. Immun. 63, 82–87. 7806387 10.1128/iai.63.1.82-87.1995 PMC172960

[B10] Guignot J. Mock M. Fouet A. (1997). AtxA activates the transcription of genes harbored by both Bacillus anthracis virulence plasmids. FEMS Microbiol. Lett. 147, 203–207. 9119194 10.1111/j.1574-6968.1997.tb10242.x

[B11] Heninger S. Drysdale M. Lovchik J. Hutt J. Lipscomb M. F. Koehler T. M.. (2006). Toxin-deficient mutants of *Bacillus anthracis* are lethal in a murine model for pulmonary anthrax. Infect. Immun. 74, 6067–6074. 10.1128/IAI.00719-06 16923785 PMC1695493

[B12] Hoffmaster A. R. Koehler T. M. (1997). The anthrax toxin activator gene *atxA* is associated with CO_2_-enhanced non-toxin gene expression in Bacillus anthracis. Infect. Immun. 65, 3091–3099. 9234759 10.1128/iai.65.8.3091-3099.1997 PMC175436

[B13] Hoffmaster A. R. Koehler T. M. (1999). Control of virulence gene expression in *Bacillus anthracis*. J. Appl. Microbiol. 87, 279–281. 10.1046/j.1365-2672.1999.00887.x 10475965

[B14] Koehler T. M. Dai Z. Kaufman-Yarbray M. (1994). Regulation of the *Bacillus anthracis* protective antigen gene: CO_2_ and a trans-acting element activate transcription from one of two promoters. J. Bacteriol. 176, 586–595. 10.1128/jb.176.3.586-595.1994 8300513 PMC205094

[B15] Leppla S. H. (1988). Production and purification of anthrax toxin. Methods Enzymol. 165, 103–116. 10.1016/S0076-6879(88)65019-1 3148094

[B16] Liang X. (1995). A Manual of Anthrax Control and Treatment. Beijing: China Agricultural Press.

[B17] Liang X. Zhang H. Zhang E. Wei J. Li W. Wang B.. (2016). Identification of the pXO1 plasmid in attenuated *Bacillus anthracis* vaccine strains. Virulence. 7, 578–586. 10.1080/21505594.2016.1164366 27029580 PMC5026784

[B18] Makino S. Uchida I. Terakado N. Sasakawa C. Yoshikawa M. (1989). Molecular characterization and protein analysis of the cap region, which is essential for encapsulation in *Bacillus anthracis*. J. Bacteriol. 171, 722–730. 10.1128/jb.171.2.722-730.1989 2536679 PMC209657

[B19] Mikesell P. Ivins B. E. Ristroph J. D. Dreier T. M. (1983). Evidence for plasmid-mediated toxin production in *Bacillus anthracis*. Infect. Immun. 39, 371–376. 6401695 10.1128/iai.39.1.371-376.1983 PMC347948

[B20] Milne J. C. Furlong D. Hanna P. C. Wall J. S. Collier R. J. (1994). Anthrax protective antigen forms oligomers during intoxication of mammalian cells. J. Biol. Chem. 269, 20607–20612. 8051159

[B21] Moayeri M. Leppla S. H. Vrentas C. Pomerantsev A. P. Liu S. (2015). Anthrax pathogenesis. Annu. Rev. Microbiol. 69, 185–208. 10.1146/annurev-micro-091014-104523 26195305

[B22] Okinaka R. T. Cloud K. Hampton O. Hoffmaster A. R. Hill K. K. Keim P.. (1999). Sequence and organization of pXO1, the large *Bacillus anthracis* plasmid harboring the anthrax toxin genes. J. Bacteriol. 181, 6509–6515. 10515943 10.1128/jb.181.20.6509-6515.1999 PMC103788

[B23] Park S. H. Oh H. B. Seong W. K. Kim C. W. Cho S. Y. Yoo C. K. (2007). Differential analysis of *Bacillus anthracis* after pX01 plasmid curing and comprehensive data on Bacillus anthracis infection in macrophages and glial cells. Proteomics 7, 3743–3758. 10.1002/pmic.200700338 17880004

[B24] Pomerantsev A. P. Sitaraman R. Galloway C. R. Kivovich V. Leppla S. H. (2006). Genome engineering in *Bacillus anthracis* using Cre recombinase. Infect. Immun. 74, 682–693. 10.1128/IAI.74.1.682-693.2006 16369025 PMC1346652

[B25] Popov S. G. Villasmil R. Bernardi J. Grene E. Cardwell J. Wu A.. (2002). Lethal toxin of *Bacillus anthracis* causes apoptosis of macrophages. Biochem. Biophys. Res. Commun. 293, 349–355. 10.1016/S0006-291X(02)00227-9 12054607

[B26] Remmele N. S. Klein F. Vick J. A. Walker J. S. Mahlandt B. G. Lincoln R. E. (1968). Anthrax toxin: primary site of action. J. Infect. Dis. 118, 104–113. 10.1093/infdis/118.1.104 5694290

[B27] Singh Y. Chaudhary V. K. Leppla S. H. (1989). A deleted variant of *Bacillus anthracis* protective antigen is non-toxic and blocks anthrax toxin action *in vivo*. J. Biol. Chem. 264, 19103–19107. 2509473

[B28] Singh Y. Klimpel K. R. Goel S. Swain P. K. Leppla S. H. (1999). Oligomerization of anthrax toxin protective antigen and binding of lethal factor during endocytic uptake into mammalian cells. Infect. Immun. 67, 1853–1859. 10085027 10.1128/iai.67.4.1853-1859.1999 PMC96537

[B29] Sirard J. C. Mock M. Fouet A. (1994). The three *Bacillus anthracis* toxin genes are coordinately regulated by bicarbonate and temperature. J. Bacteriol. 176, 5188–5192. 10.1128/jb.176.16.5188-5192.1994 8051039 PMC196368

[B30] Smith H. Keppie J. (1954). Observations on experimental anthrax; demonstration of a specific lethal factor produced *in vivo* by *Bacillus anthracis*. Nature 173, 869–870. 10.1038/173869a0 13165673

[B31] Stanley J. L. Smith H. (1961). Purification of factor I and recognition of a third factor of the anthrax toxin. J. Gen. Microbiol. 26, 49–63. 10.1099/00221287-26-1-49 13916257

[B32] Uchida I. Hashimoto K. Terakado N. (1986). Virulence and immunogenicity in experimental animals of *Bacillus anthracis* strains harbouring or lacking 110 MDa and 60 MDa plasmids. J. Gen. Microbiol. 132, 557–559. 10.1099/00221287-132-2-557 3086499

[B33] Uchida I. Hornung J. M. Thorne C. B. Klimpel K. R. Leppla S. H. (1993). Cloning and characterization of a gene whose product is a trans-activator of anthrax toxin synthesis. J. Bacteriol. 175, 5329–5338. 10.1128/jb.175.17.5329-5338.1993 8366021 PMC206586

[B34] Uchida I. Makino S. Sekizaki T. Terakado N. (1997). Cross-talk to the genes for *Bacillus anthracis* capsule synthesis by atxA, the gene encoding the trans-activator of anthrax toxin synthesis. Mol. Microbiol. 23, 1229–1240. 10.1046/j.1365-2958.1997.3041667.x 9106214

[B35] Welkos S. L. Vietri N. J. Gibbs P. H. (1993). Non-toxigenic derivatives of the Ames strain of *Bacillus anthracis* are fully virulent for mice: role of plasmid pX02 and chromosome in strain-dependent virulence. Microb. Pathog. 14, 381–388. 10.1006/mpat.1993.1037 8366815

[B36] Yang J. Liang X. Ji Y. (2015). The novel transcriptional regulator SA1804 Is involved in mediating the invasion and cytotoxicity of *Staphylococcus aureus*. Front. Microbiol. 6:174. 10.3389/fmicb.2015.00174 25806024 PMC4353350

[B37] Yeager L. A. Chopra A. K. Peterson J. W. (2009). *Bacillus anthracis* edema toxin suppresses human macrophage phagocytosis and cytoskeletal remodeling via the protein kinase A and exchange protein activated by cyclic AMP pathways. Infect. Immun. 77, 2530–2543. 10.1128/IAI.00905-08 19307216 PMC2687349

