# Supplementary figures and images for: The pag Gene of pXO1 Is Involved in Capsule Biosynthesis of Bacillus anthracis Pasteur II Strain
Source: Front Cell Infect Microbiol. 2017 May 26;7:203. doi: 10.3389/fcimb.2017.00203 (PMC5445325; doi:10.3389/fcimb.2017.00203)

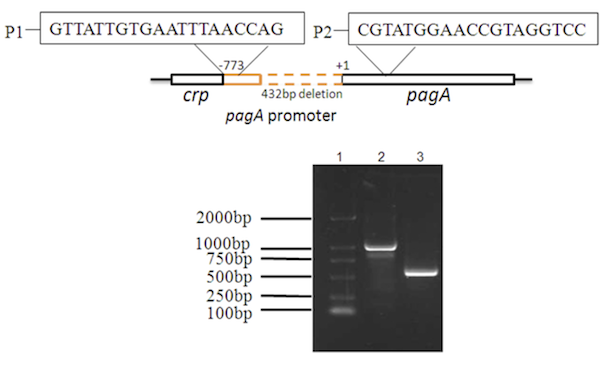

Supplement: Supplementary Figure 1 — The deletion of pag gene promoter as indicated by PCR. Lane 1: DNA molecular marker. Lane 2: PasteurII, and Lane 3: PasteurII- pag promoter-KO. [file Image1.TIFF]

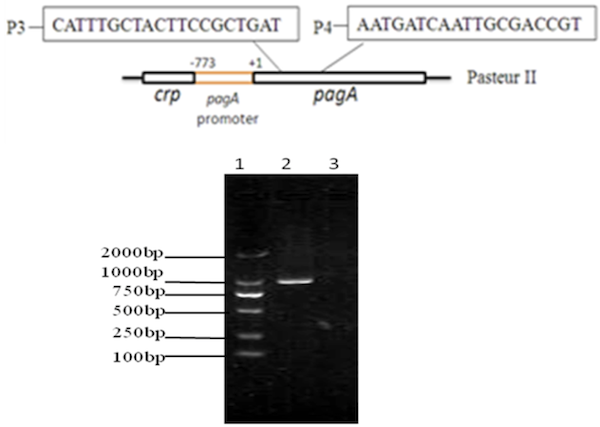

Supplement: Supplementary Figure 2 — The deletion of pag gene as indicated by PCR. Lane 1: DNA molecular marker. Lane 2: PasteurII, and Lane 3: PasteurII- pag KO. [file Image2.TIFF]
